# Anterior cement augmentation technique for correction of kyphotic deformity in the lumbar spine using an anterior-to-Psoas approach

**DOI:** 10.1016/j.xnsj.2025.100818

**Published:** 2025-10-29

**Authors:** David Shin, David Cheng, Debra Cheng, Carson Cummings, Jacob Razzouk, Olumide Danisa, Wayne Cheng

**Affiliations:** a Loma Linda University School of Medicine, 11175 Campus Street, Loma Linda, CA 92350, United States; b Bones and Spine Surgery, 25915 Baron Road #203, Loma Linda, CA 92354, United States; c Departments of Orthopaedic Surgery and Neurological Surgery, Duke University Health System, Durham, NC, United States

**Keywords:** Kyphosis, Cement, Anterior cement augmentation, Kyphotic deformity, Lumbar

## Abstract

**Background:**

Posterior kyphotic deformity correction in patients with osteoporosis carries a significant risk of instrumentation cut-out. Anterior approaches allow direct distraction at the center of the deformity, yet reduction devices or instrumentation may telescope into vertebral endplates. This study proposes an anterior cementing augmentation technique for correction of kyphotic deformity involving a less invasive oblique lateral interbody fusion (OLIF) approach.

**Case description:**

Patient 1 had a previous compression fracture at L2 above prior instrumented fusion, with kyphotic deformity, and underwent a stage-one anterior lateral L2 corpectomy via an (OLIF) approach. Cement was placed centrally at L1 and L3, followed by an expandable cage and a stage-two posterior T11-to-pelvis instrumented fusion with prophylactic cement augmentation at T10–T12. Patient 2 had a previous remote L5–S1 anterior lumbar interbody fusion and underwent a posterior C4–S1 osteotomy and instrumented fusion for kyphoscoliosis deformity. The patient developed immediate postoperative instrumentation failure with kyphosis at L4–L5 and instrumentation loosening and migration at L4–S1 within 2 weeks of surgery. They then underwent (OLIF) at L4–L5 with anterior cement augmentation at L4–L5, followed by a revision lumbar-pelvic instrumented fusion.

**Outcome:**

A step-by-step technique was described for anterior corpectomy and cement augmentation via an anterior lateral approach, presenting an alternative for correction of kyphotic deformity and restoration of sagittal alignment in the lumbar spine of patients with poor-bone density.

**Conclusions:**

A minimally invasive, anterior-to- psoas approach may decrease morbidity of approach related complications. Both patients demonstrated kyphotic deformity correction with follow-up records suggesting favorable outcomes.

## Introduction

Osteoporosis affects more than 10.3 million Americans and predisposes individuals to an increased risk of vertebral fractures and worsening kyphotic deformity [[1], [2], [3]]. Surgical correction of kyphotic deformity requires preoperative assessment for bone density and quality, commonly determined via dual energy X-ray absorptiometry (DEXA) [1]. Kyphotic deformity correction via a posterior approach in patients with osteoporosis or osteomalacia carries significant risk of instrumentation cut-out and inadequate correction. Anterior approaches have advantages of direct distraction at the center of the deformity, yet reduction devices or instrumentation may telescope into vertebral endplates. Surgical techniques that can minimize risks associated with osteoporosis while simultaneously correcting kyphotic deformity are of high clinical value [1,4]. Although cement augmentation and oblique lateral interbody fusion (OLIF) techniques have been individually explored and established, this study proposes a novel anterior cement augmentation technique for correction of kyphotic deformity involving an OLIF approach. The technique supports the apex of the kyphosis and allows for the ability to provide anterior column support in patients with poor bone quality and density utilizing a less invasive approach.

## Case presentation

Following IRB approval (#5240053), we analyzed two cases that presented with lumbar kyphotic deformity and poor bone quality.

### Case one

**Fig. 1:**
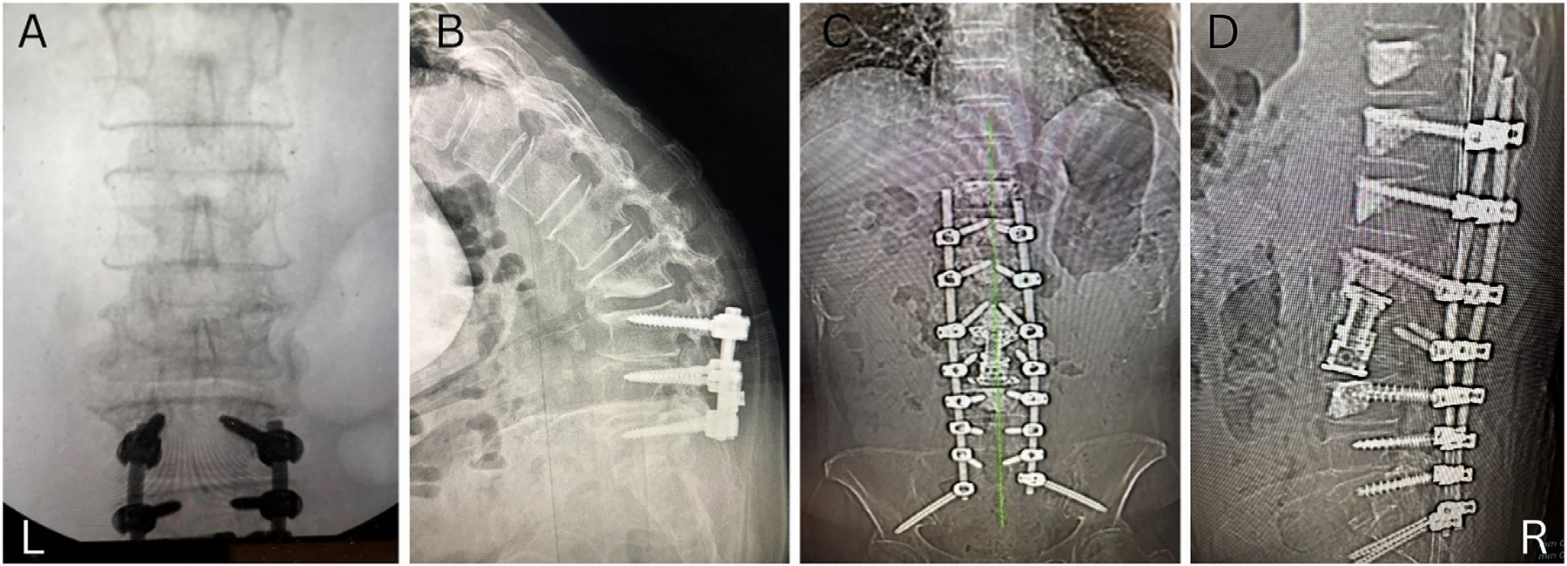
A. Preoperative anteroposterior radiograph demonstrating kyphosis above the fusion. B. Preoperative lateral radiograph demonstrating kyphosis above the fusion. C. postoperative anteroposterior radiograph demonstrating kyphosis correction. D. Postoperative lateral radiograph demonstrating kyphosis correction.

The first patient was a frail 70-year-old female with a body mass index (BMI) of 25.6 kg/m^2^ (1.57 m, 63.5 kg) and a DEXA T-score of −3.1. The patient presented to our clinic 1 year after a L3–L5 fusion with complaints of lower back pain (9/10) and the inability to stand. The patient developed an L2 compression fracture in an attempt to stand the first day after her initial lumbar fusion. Her past medical history was significant for type II diabetes mellitus, history of gastric cancer and chemotherapy, and hypertension. Supine radiographs demonstrated transitional anatomy, her lumbar lordosis was negative 36°, sagittal vertical axis was positive 33 cm, pelvic incidence was 40°, and pelvic tilt was 30°. Fig. 1A and B show anteroposterior (AP) and lateral preoperative radiographs demonstrating the kyphotic deformity above a spinal fusion.

The patient underwent a stage-one procedure including an anterior lateral L2 corpectomy via an oblique lateral interbody fusion approach. Cement was placed anterolaterally at L1 and L3. An expandable cage (Medtronic, Memphis, TN) was placed. A stage-two procedure was performed 2 days later with posterior T11 to pelvis instrumented fusion with cement augmentation at 10 to T12. AP and lateral radiographs demonstrating the final postoperative construct are shown in Fig.1C and D. A full description of our surgical technique is described below.

### Patient positioning and exposure

Patients were placed in a right lateral decubitus position with their left side up on a flat spine Jackson table. AP and lateral fluoroscopic images were used to ensure a true orthogonal position to the fluoroscope.

**Fig. 2:**
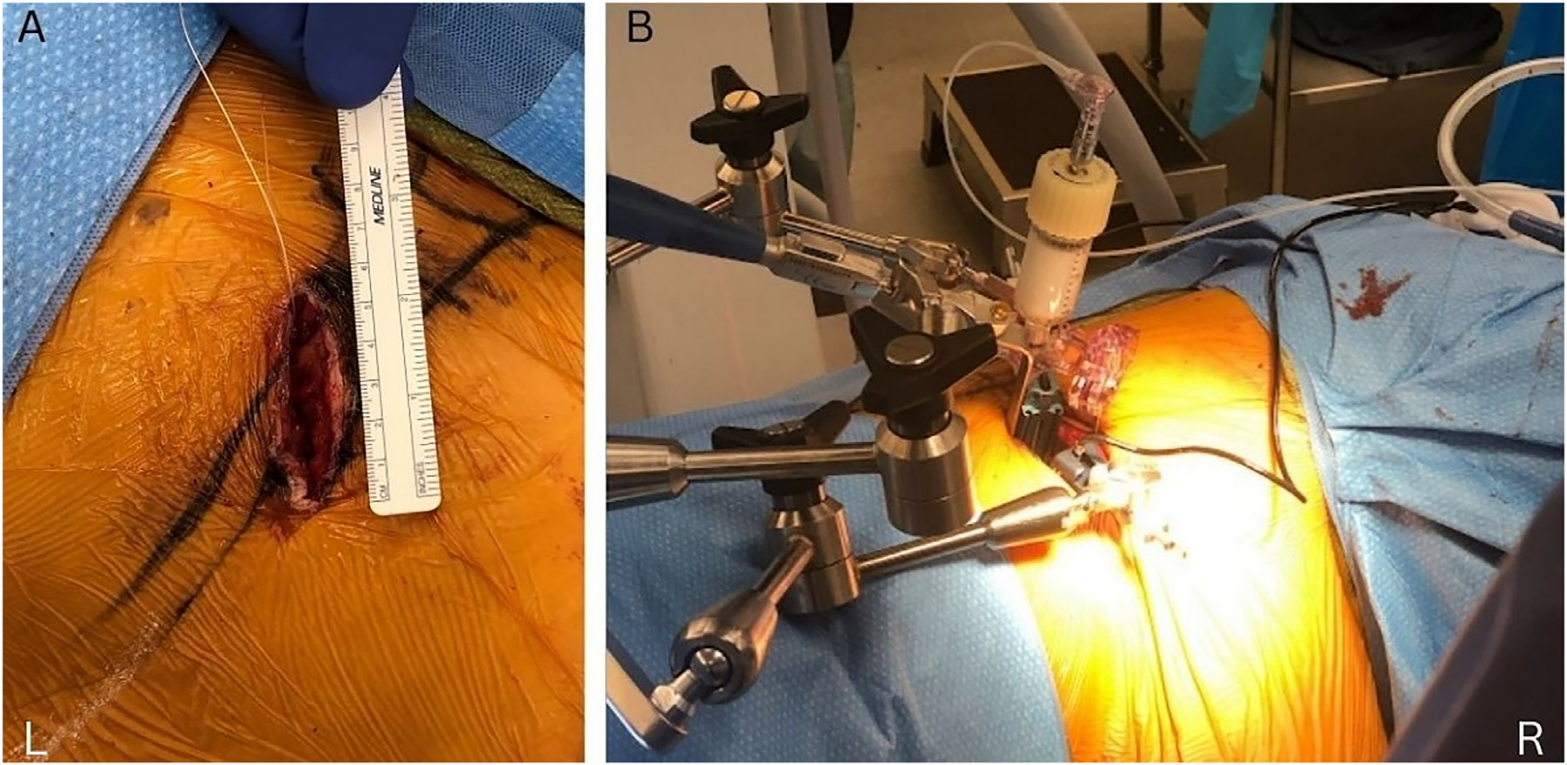
A. Incision demonstrating minimally invasive technique. B. Setting up cement for injection.

A mini open incision was made approximately 3 finger breaths in front of the vertebral body. Three layers of abdominal musculature were identified and bluntly dissected in the direction of the muscle fibers. Fig. 2A illustrates this minimally invasive incision. The retroperitoneal space is developed between the great vessels and psoas muscle, and the retractor (Medtronic OLIF, Memphis Tenn.) was docked on the most kyphotic vertebrae (Fig. 2B).

### Needle and cement placement

**Fig. 3:**
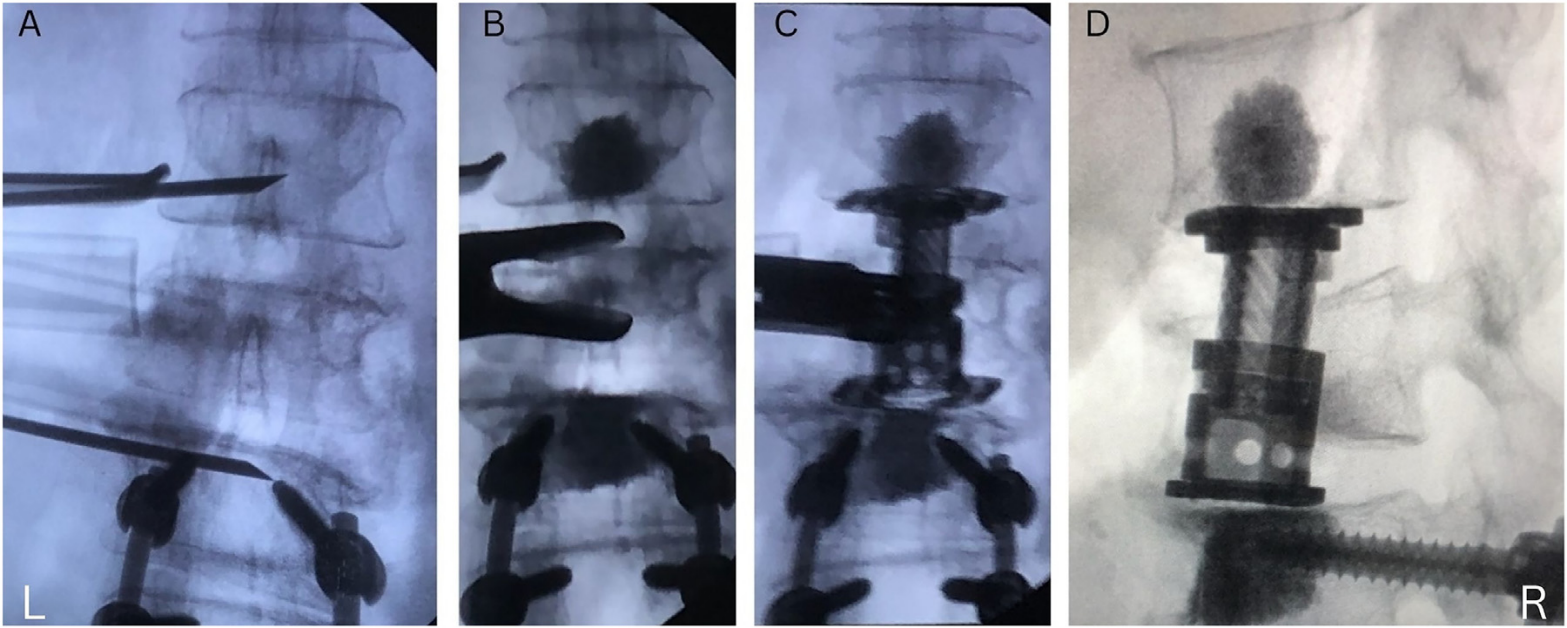
A. Anteroposterior view demonstrating lateral needle placement in the midline above and below endplates. B. Corpectomy after cement augmentation. C. Anteroposterior view of expandable cage for corpectomy site after anterior cement augmentation. D. Lateral view of expandable cage for corpectomy site after anterior cement augmentation.

Fig. 2B showcases setting up the cement for injection. A 17.5-cm, 8-gauge needle was placed above and below the end plates, adjacent to the most kyphotic vertebrae, illustrated by Fig. 3A. The needle must be advanced to the midline of the vertebrae on AP fluoroscopic images to prevent cement obstructing future posterior screw advancement. Additionally, the needle must be placed in the anterior portion of the vertebral body, confirmed on lateral fluoroscopic images, to prevent cement extrusion into the spinal canal. 1–3 cc of doughy cement (Depuy Synthes Confidence Spinal Cement, Raynham MA) was injected slowly into the vertebral body visualized by lateral fluoroscopic images.

### Corpectomy and deformity correction

Discectomy and corpectomy were performed in standard fashion, with Fig. 3B showcasing the corpectomy of L2 after cementation of the end plate of L1 and L3. The top and bottom end plates of the expandable cage should be wide enough to cross close to both sides of the apophyseal ring. The cage should be placed at the midline and anterior ⅓ of the vertebral body at the corpectomy site. Expansion of the cage is based on the relative height of the level above and below the level of kyphosis. A morselized vertebral body bone graft was placed around the cage. An AP and lateral view of the expandable cage for corpectomy site after anterior cement augmentation are shown by Fig. 3C and D.

### Posterior instrumentation with augmentation

Either an open or percutaneous screw should be placed posteriorly. Posterior cement augmentation is usually needed before compression and deformity correction. Augmentation above and below the end instrumented vertebrae should be considered if longer construction is desired.

A stage-two procedure was performed 2 days following stage one. A posterior T12-to-pelvis instrumented fusion was performed with prophylactic cement augmentation at T11, followed by T12, L1, and L2 vertebral body augmentation. Pontine osteotomy and compression were performed and applied between L1 and L3.

### Case two

**Fig. 4:**
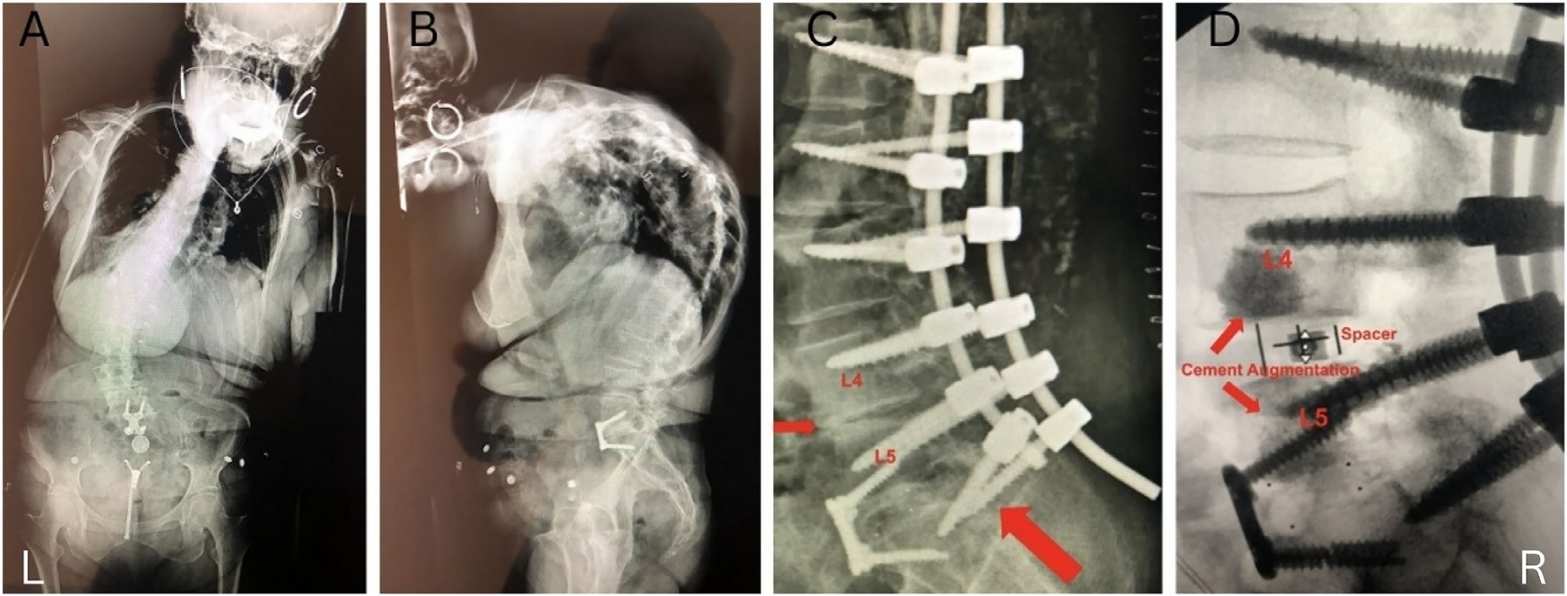
A. Anteroposterior preoperative radiograph demonstrating kyphoscoliosis. B. Lateral preoperative radiograph demonstrating kyphoscoliosis. C. Lateral radiograph demonstrating loosening and migration of L5, S1 pedicle screws and disc collapse and kyphosis at L4–L5. D. Lateral intraoperative radiograph demonstrating cement and spacer placement.

**Fig. 5:**
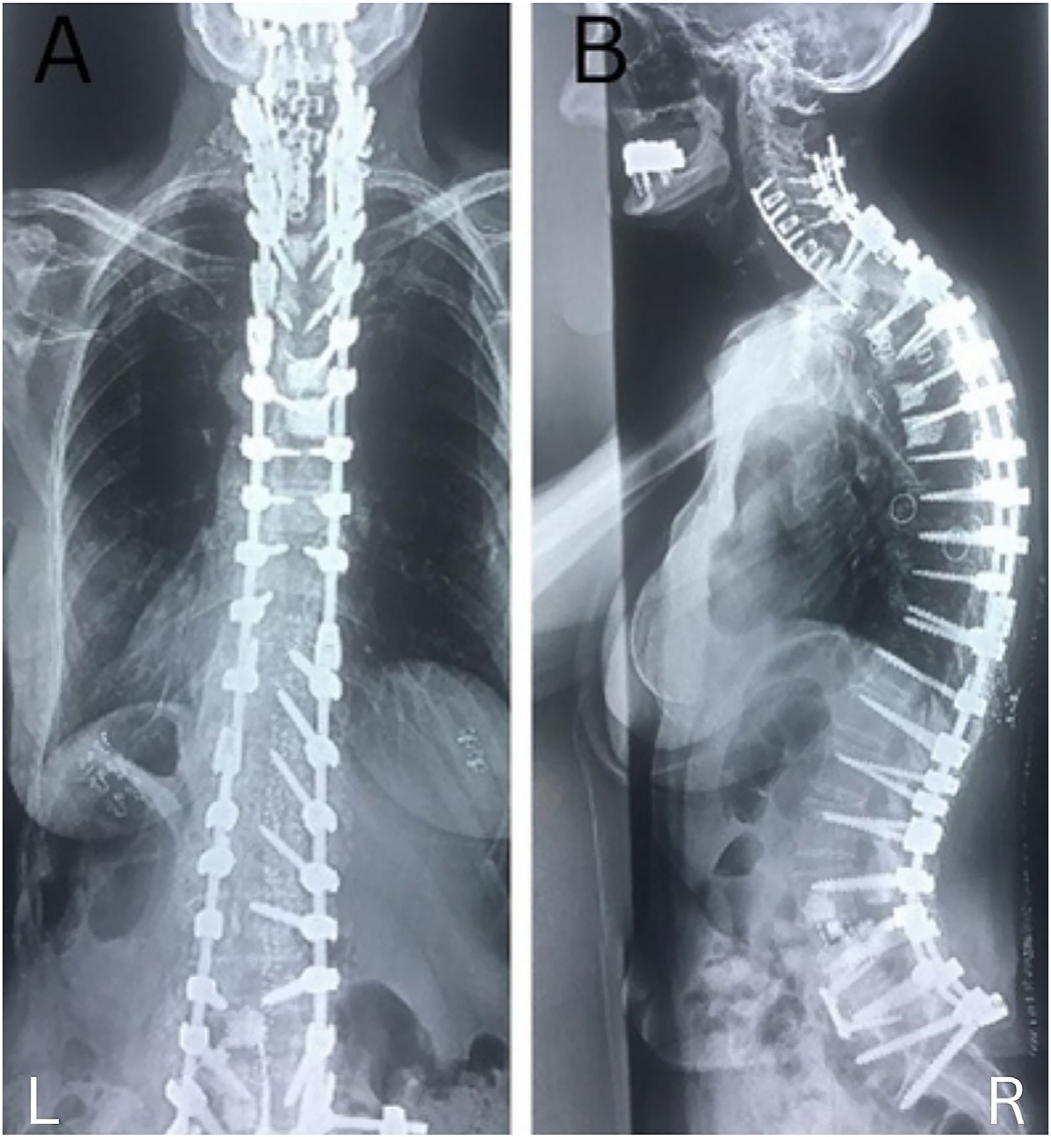
A. Anteroposterior radiographs demonstrating final postoperative construct. B. Lateral radiographs demonstrating final postoperative construct.

The second patient was a 66-year-old female with BMI of 18.21 kg/m^2^, (1.67 m, 50.8 kgs) and DEXA T-score of −1.2. The patient reported no significant medical history but had a surgical history of anterior lumbar interbody fusion (ALIF) at L5–S1 approximately 19 years ago and presented to our clinic with kyphoscoliosis. Fig. 4A and B illustrate AP and lateral radiographs of her kyphoscoliosis. We performed a posterior C4–S1 osteotomy and instrumented fusion. Intraoperatively the patient had extremely poor bone quality with low insertional torque during instrumentation, despite a normal DEXA T-score [5]. The patient developed immediate postoperative instrumentation failure with kyphosis at L4–L5 and instrumentation loosening and migration at L4–S1 within 2 weeks of surgery (Fig. 4C). The patient then underwent oblique lateral interbody fusion at L4–L5 with anterior cement augmentation, followed by a revision lumbar-pelvis instrumentation (Fig. 4D). AP and lateral radiographs demonstrating the final postoperative construct are shown in Fig. 5A and B.

## Discussion

Current treatments for kyphotic patients with concurrent osteoporosis often utilize pharmacotherapeutic intervention followed by surgery. Options include antiresorptive agents such as bisphosphonates or parathyroid hormone (PTH) analogs such as teriparatide [6]. PTH analog treatment has demonstrated effectiveness in improving surgical outcomes, but treatments involve months to years of usage, vary in their availability based on insurance, and often inadequately improve bone density [7,8]. Consequently, this relatively marginal improvement may be unsatisfactory to qualify severely, or even moderately, osteoporotic patients for surgical candidacy.

Both patients presented with significant deformity, poor bone quality, and postoperative complications from a prior surgery for spinal deformity. Patient 1 demonstrated a DEXA T-score of −3.1, indicating osteoporosis, while patient 2 possessed a DEXA T-score of −1.2 [5]. While this T-score did not indicate osteoporosis, it was evident intraoperatively that the patient possessed extremely poor bone quality. This highlights the reality that DEXA values are not always congruent with what is empirically experienced, with DEXA reflecting the quantity of bone but not always the quality of bone [9]. DEXA quantifies bone mass but does not assess microarchitectural integrity, cortical thickness, or trabecular connectivity, all of which affect implant fixation and resistance to subsidence [10]. Possible etiologies for the discrepancy include disuse osteopenia from longstanding spinal deformity, prior lumbar fusion with altered load distribution, or underlying metabolic changes not captured by DEXA [[11], [12], [13]]. These factors suggest that patients with “normal” T-scores may still benefit from cement augmentation when intraoperative bone quality is poor.

Our choice in cementation and surgical technique was influenced by the patients’ frailty, poor bone quality, and the goal of minimizing additional exposure and approach-related morbidity. However, the authors acknowledge that additional structural factors such as fixation strategy may contribute to instrumentation failure. Sound biomechanical planning, such as selecting cages with broader endplate contact surfaces, in addition alternative fixation methods such as Harms cages, pelvic fixation, and rod contouring should be considered when appropriate in revising and preventing instrumentation failure of fusion constructs.

Specific techniques of kyphoplasty are often considered on a case-by-case approach, with a posterior approach for cement augmentation most frequently utilized [14]. Posterior cement augmentation is effective in preventing postoperative instrumentation failure, with many surgeons performing posterior instrumentation on neighboring vertebrae to prevent fractures [15]. However, adequate deformity correction is difficult to achieve with posterior-only instrumentation in osteoporotic bone [16]. Anterior-based surgical interventions offer certain advantages in safety, as well as direct correction of kyphotic deformity at its apex [[16], [17], [18]]. Adding an intervertebral body device such as a cage, could subside in poor quality bones during kyphosis correction. Cement augmentation alone does not correct kyphosis, but when performed prior to the insertion of a cage, it significantly decreases the chance of subsidence. This improves kyphotic correction during anterior cage insertion and allows the cage to be used as a fulcrum during posterior kyphosis correction using segmental compression maneuvers. Anterior cementing techniques have been previously described as part of an ALIF technique for spondylolisthesis [19]. Biomechanical studies have demonstrated that anterior cement augmentation of adjacent levels after vertebral body replacement leads to superior stability of the corpectomy cage under cyclic loading [17].

Our anterior cement augmentation technique offers advantages with the ability to place cement close to the endplate, which may provide immediate local support where subsidence typically occurs during corpectomy with expandable cages. This targeted support has the potential to reduce the risk of cage subsidence, which remains one of the main limitations of using expandable cages in poor bone quality. Compared with traditional ALIF or open corpectomy approaches, this OLIF technique also allows for a minimally invasive, anterior-to-psoas pathway that reduces direct psoas muscle morbidity, avoids transperitoneal dissection, and requires a smaller incision. This corridor provides direct access to the anterior vertebral body for cement placement while minimizing vascular and visceral mobilization. We recognize that similar anterior cement augmentation techniques may have been performed by other surgeons in various contexts, with many components of this procedure having precedent in literature. However, the variation of technique detailed in this study is the first utilization of a minimally invasive OLIF for lumbar kyphosis correction in patients with poor bone quality.

### Limitations

Despite the benefits, this technique may not be suitable for all lumbar kyphotic deformities. Possible disadvantages of cement emboli, the risk of adjacent compression fractures, and approach-related hazards to proximal anatomical structures have not been fully examined. Further investigation with a larger population should be performed, considering factors such as location and severity of the deformity. An additional limitation of this report is the absence of long-term postoperative imaging beyond the immediate postoperative period. While clinical follow-up was available for both patients, with both patients reporting significant improvement in back pain and functional status, longer-term radiographic outcomes were unavailable. Despite follow-up records suggesting favorable outcomes, the durability of subsidence prevention and long-term construct stability cannot be fully assessed. However, we believe the intraoperative technical feasibility, perioperative safety, and immediate correction achieved highlights the potential utility of this approach. Future work should focus on prospective collection of clinical and radiographic follow-up to validate the durability of this surgical approach. Additionally, comparative studies with other established techniques for kyphotic correction will be valuable in assessing the longevity and potential complications associated with this technique.

## Conclusion

Our anterior cement augmentation technique presents a novel alternative for correction of kyphotic deformity and restoration of sagittal alignment in the lumbar spine, offering the advantage of performing aggressive deformity correction in poor quality bone. Using a minimally invasive, anterior-to-psoas approach may decrease morbidity of approach related complications. Follow-ups were conducted with favorable outcomes, but further investigation is warranted to assess the risks-to benefits profile of this technique.

## Data Availability Statement

The data that support the findings of this study are not publicly available due to information that could compromise the privacy of research participants but are available upon reasonable request.

## Ethical approval

Informed consent was not required for this study as all data used was de-identified before analysis. This decision was made by the Loma Linda University Health Institutional Review Board (IRB) #5240053 which deemed the project exempt.

## Patient informed consent statement

Complete written informed consent was obtained from the patient for the publication of this study and accompanying images.

## Declaration of competing interest

The authors declare that they have no known competing financial interests or personal relationships that could have appeared to influence the work reported in this paper.
